# Roles and thresholds of viscosity and interfacial tension in surfactant flooding for residual oil recovery

**DOI:** 10.3389/fchem.2025.1660041

**Published:** 2025-09-18

**Authors:** Jiangtao Wang, Yingxue Hu, Xiaoyue Chu, Gangzheng Sun, Tao Lu

**Affiliations:** 1 School of Human Settlement and Civil Engineering, Xi’an Jiaotong University, Xi’an, China; 2 School of Chemistry, Xi’an Jiaotong University, Xi’an, China; 3 Research Institute of Petroleum Engineering and Technology, Shengli Oilfield Company, Sinopec, Dongying, China; 4 Shaanxi Detian Licheng New Materials Technology Co., Ltd., Xi’an, China

**Keywords:** surfactant flooding, enhanced oil recovery, viscosity, interfacial tension, wettability

## Abstract

Surfactant flooding is an effective chemical enhanced oil recovery (EOR) technique, but the quantitative roles of viscosity and interfacial tension (IFT) in residual oil mobilization remain unclear. In this study, the physicochemical properties of surfactant solutions were first characterized by systematic measurements of viscosity and IFT, and the dynamic mechanisms of water flooding and surfactant flooding were further investigated using a pore–throat model, consisting of channels with and without pore-like structures, combined with direct numerical simulations to identify viscosity and IFT thresholds under different wettability conditions. The results show that residual oil distribution is strongly influenced by wettability: in strongly water-wet and neutral-wet conditions, oil remains as droplet- or cluster-like ganglia within pore spaces, whereas in strongly oil-wet conditions it persists as continuous wall-adhered films that are more stable and difficult to mobilize. Mechanistic analysis further indicates that the controlling parameters of mobilization differ across wettability states, reflecting the interplay between pore geometry, wettability, and interfacial forces. Based on phase diagrams constructed from simulation results, distinct regulation strategies were formulated for different wettability conditions: reducing IFT is most effective in strongly water-wet systems, a combined effect of viscosity enhancement and IFT reduction is required in neutral-wet systems, and achieving ultra-low IFT is essential in strongly oil-wet systems. Collectively, this work establishes viscosity and IFT thresholds as quantitative design criteria for surfactant flooding, providing both mechanistic understanding and practical guidance for surfactant formulation and injection optimization in chemical EOR.

## 1 Introduction

As a commonly used chemical flooding method for enhanced oil recovery, surfactant flooding has been widely used in the petroleum industry (Bashir et al., 2022; Demirbas et al., 2015; Druetta and Picchioni, 2018; Hirasaki et al., 2011; Qu et al., 2023). Generally, the two fundamental ways to enhance oil recovery are to expand the swept volume and increase the oil displacement efficiency. Surfactants are amphiphilic molecules with hydrophilic head groups and hydrophobic tail groups (Keshtkar et al., 2016). The method of using surfactant solution as a displacement agent to enhance the crude oil recovery is surfactant flooding technology. By injecting surfactant into reservoirs, the oil-water interfacial tension can be reduced (Hematpour et al., 2012; Lv et al., 2014; Zhu et al., 2013), and the permeability of crude oil in the pores can be improved, thereby enhancing the crude oil recovery (Chen et al., 2021). In recent years, the development of surfactants is varied, and surfactants have developed viscoelastic surfactants with the ability of reducing tension and increasing viscosity. Therefore, in surfactant flooding, mobility ratio and capillary number will be affected. It can better drive the trap fluid and recover the remaining oil by reducing IFT. Residual oil refers to the immobile oil remaining in the reservoir after conventional water flooding, mainly trapped by capillary forces and pore structure heterogeneity. In recent years, surfactant systems have diversified to include not only viscoelastic surfactants with dual functionalities (Gbadamosi et al., 2019), but also middle-phase microemulsion formulations capable of achieving ultra-low IFT and effectively mobilizing trapped oil in low-permeability and conservational reservoirs (Kokkinos et al., 2022; Liu et al., 2017; Wu et al., 2024; Yousefrooz et al., 2025).

According to the theory of capillary number, oil displacement efficiency is closely related to parameters such as displacement velocity, viscosity, interfacial tension, etc. Some researchers have also studied the impact of interfacial tension on oil recovery. In 1927, Uren and Fahmy pointed out that the efficiency of water flooding is inversely proportional to the interfacial tension of the displacing fluid in crude oil production (Uren and Fahmy, 1927). In 1966, Wagner and Leach, (1966) found from their experimental results that the oil displacement efficiency can be increased only at the interfacial tension below 0.07 mN/m and thus significantly increased by further reducing the interfacial tension. Melrose, (1974) believed that in order to enhance oil recovery, ultra-low oil-water interfacial tension is required. Zhao et al. (2006) believed that in order to displace oil in reservoir rock pores and capillaries, the interfacial tension between crude oil and water slugs containing surfactants must be reduced to an ultra-low level (<10^–2^ mN/m). In these studies, surfactant formulations with different IFT levels were obtained by changing the concentration or type of surfactant and used to investigate the impact of IFT on oil recovery (Jinmei et al., 2025; Pu et al., 2016). Capillary force is the cause of fluid occlusion during immiscible displacement in porous media. Laboratory research shows that if the viscosity increased, more residual oil can be recovered in immiscible displacement. By reducing capillary force, trapped residual oil may be produced.

The displacing fluid with higher viscosity has stronger ability to expand the swept volume and better oil displacement effect. However, with increasing viscosity above a certain level, its ability to improve the oil displacement effect deteriorates. Therefore, the displacing fluid must have a reasonable upper threshold value of viscosity (Demin et al., 2011). Currently, surfactant flooding is the most complex and therefore the most uncertain. If the surfactant formula used for oil recovery is properly designed and its flow in the reservoir is appropriately controlled, it has high potential to achieve maximum oil recovery (Guo et al., 2017). In oil production, the efficiency of oil recovery strongly depends on the wettability of the reservoir rock (Mungan, 1964). The interaction between capillary forces and viscous forces varies with different wettability conditions (Johannesen and Graue, 2007; Zhou et al., 1996). Moreover, the effectiveness of surfactants in reducing interfacial tension is significantly influenced by reservoir wettability.

In practical applications, EOR by surfactant flooding is uncertain due to the complex action mechanism of surfactant and the complex geological structure of reservoirs. In order to better understand the surfactant flooding mechanism and achieve optimal application effect, a new theoretical model for residual oil migration was proposed in this study. This model takes the combined effects of interfacial tension and viscous force as well as the effects of wettability, opening angle of pore structure, pore size distribution, etc. We used this model for the concept of parallel capillaries, where fluid flows through two channels, one of which contains pores and the other does not, to simulate water flooding. The direct numerical simulations method based on the Navier Stokes Equation and Volume of Fluid (VOF) method was used to study the effects of viscosity and interfacial activity on the residual oil production ability in the formations with different wettability. In addition, we studied the mechanism of EOR by displacing heavy oil with different viscosities using different chemical systems, and determined the influence of mobility ratio on input-output ratio as well as the viscosity ratio threshold. We further determined the law of enhancing the heavy oil recovery by water flooding under the combined action of viscosity and interfacial activity as well as the viscosity ratio threshold. These research results are of great significance for understanding the surfactant flooding mechanism, achieving optimal displacing effects, and enhancing the oil recovery.

## 2 Surfactants properties

### 2.1 Types of surfactants

**FIGURE 1:**
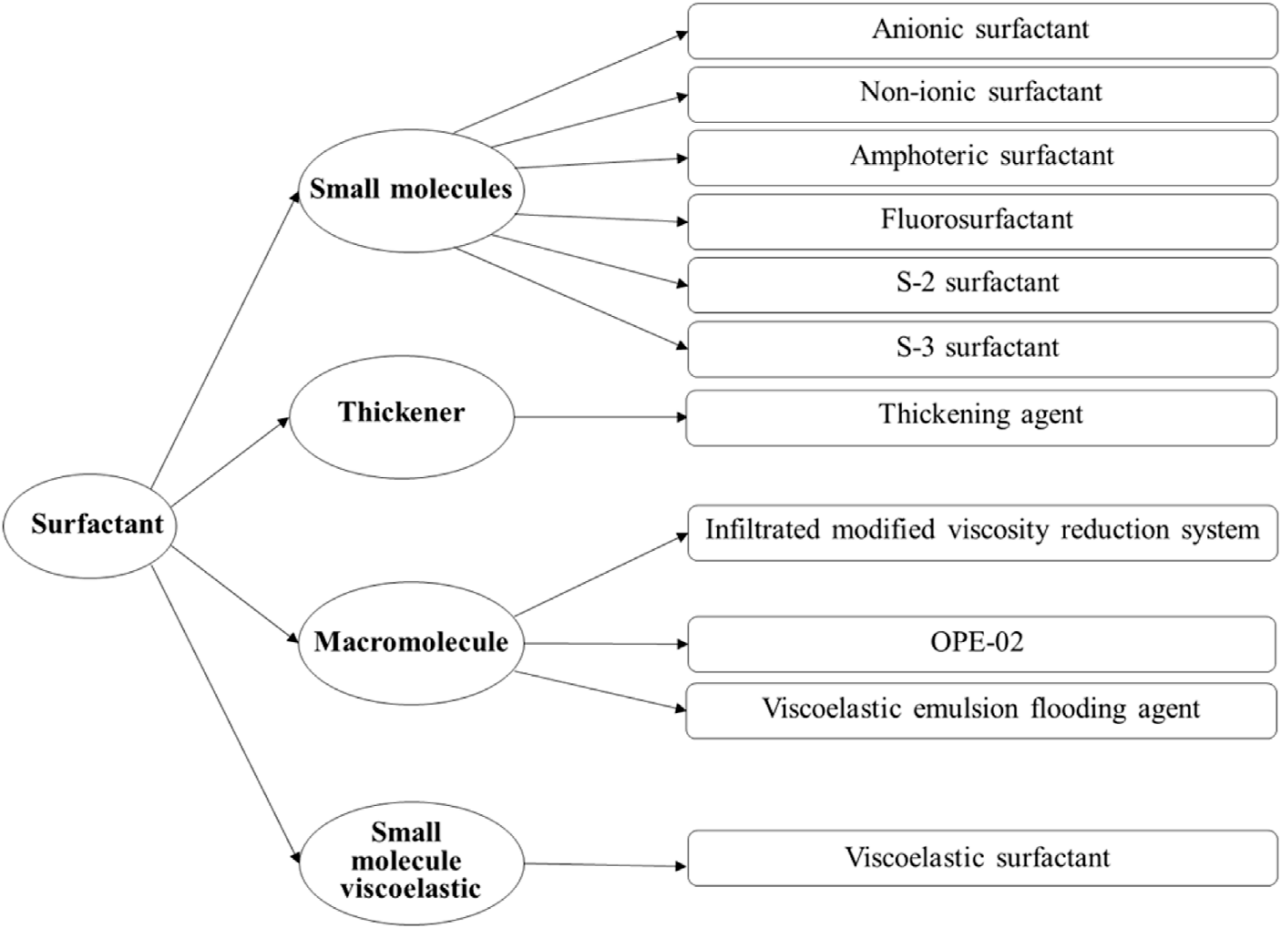
Classification of surfactants used in the experiment. The classification is based on molecular structure and rheological functionality, distinguishing between small molecule surfactants, thickeners, polymer surfactants, and viscoelastic surfactants.

In order to elucidate the patterns of EOR during water flooding of viscous oil under the synergistic influence of viscosity and interfacial activity, as well as to determine the thresholds of viscosity and interfacial tension, eleven distinct types of surfactants were systematically chosen. The detailed classification is illustrated in Figure 1, presenting the taxonomy of the eleven surfactants employed in the experimental investigation. Based on their respective interfacial activity and viscosity profiles, these surfactants were categorized into four classes: small molecule surfactants (including anionic, non-ionic, amphoteric, fluorinated, S-2, and S-3 surfactants), thickeners, polymer surfactants (comprising permeability-modifying viscosifying agents, viscoelastic emulsified displacement agents, and OPE-02 polymer surfactants), and synthetically crafted small molecule viscoelastic surfactants.

The agents utilized in the experiments, namely, the permeability-modifying viscosifying agent and viscoelastic emulsified displacement agent, as well as five additional agents (S-2 surfactant, S-3 surfactant, OPE-02 polymer surfactant), were sourced from the Shengli Oilfield. The thickeners (polyacrylamide), anionic surfactants, non-ionic surfactants, amphoteric surfactants, and fluorinated surfactants were procured from Aladdin, while the viscoelastic surfactant was synthesized in-house within the laboratory. This systematic classification and procurement strategy underscore the rigorous approach undertaken in the experimental design for the investigation of surfactant-induced EOR.

### 2.2 Measurement of viscosity and interfacial tension

Chemical flooding dynamics and displacement are influenced by multiple factors, including interfacial tension, viscosity ratio, and flow velocity (Liu and Shen, 2015). The viscosity and interfacial tension of the surfactant solutions were measured using a Pro + DV-3 digital viscometer (Shanghai Nirun Intelligent Technology Co., Ltd., China) and a Krüss K100 force tensiometer (Krüss GmbH, Germany), respectively. All measurements were conducted at a constant temperature of 25 °C. Viscosity was measured at a fixed rotational speed of 60 rpm and reported in mPa·s. Interfacial tension was determined using the Du Noüy ring method, employing a platinum–iridium ring calibrated before each test. Surfactant solutions were prepared with deionized water, and kerosene was used as the oil phase. Each measurement was repeated three times, and the average value was used. The results were obtained with an accuracy of ±0.1 mPa s for viscosity and ±0.01 mN/m for interfacial tension. These standardized procedures ensured the reliability and reproducibility of the physicochemical characterization of the surfactants.

**FIGURE 2:**
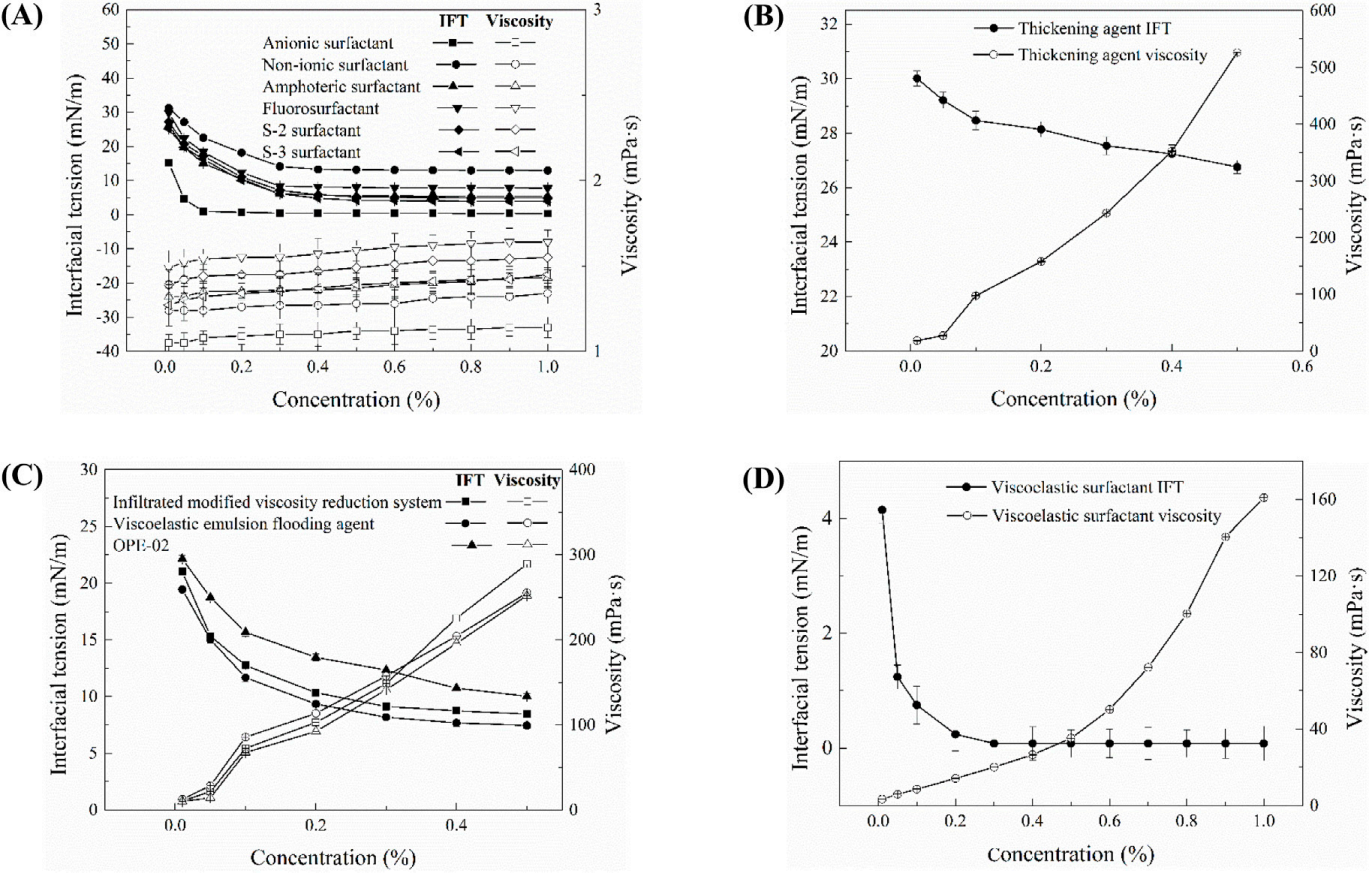
Viscosity and interfacial tension of surfactants: **(A)** small molecule surfactants; **(B)** thickeners; **(C)** high-molecular-weight surfactants; **(D)** viscoelastic surfactants. The classification is based on molecular structure and rheological functionality.

Figure 2 depicts the interfacial tension and viscosity characteristics of 11 selected chemical agents at varying concentrations, with interfacial tension measured against kerosene. As shown in Figure 2A, the viscosity of six small-molecule surfactants remains nearly constant, approximating the viscosity of water, even as concentration increases. The interfacial tension between these surfactants and kerosene consistently decreases within the concentration range of 0.01 wt% to 0.3 wt%. In the range of 0.3 wt% to 1.0 wt%, the interfacial tension stabilizes, demonstrating a decreasing efficacy order of anionic surfactant, S-3 surfactant, amphoteric surfactant, S-2 surfactant, fluorinated surfactant, and nonionic surfactant. Figure 2B elucidates the viscosity and interfacial tension of thickening agents, indicating a marked increase in viscosity with concentration, although their capacity to reduce interfacial tension with kerosene is relatively subdued.

Figure 2C presents the viscosity and interfacial tension of high-molecular-weight surfactants. Within the concentration range of 0.01 wt% to 0.3 wt%, these surfactants exhibit a pronounced reduction in interfacial tension as concentration increases. Between 0.3 wt% and 0.5 wt%, the reduction trend becomes more gradual. The IFT reduction efficiency follows the order: viscoelastic emulsifying displacement agent > permeability-modifying viscosity-reducing agent > OPE-02 surfactant. All three high-molecular-weight surfactants exhibit a significant and similar increase in viscosity as concentration increases.

Figure 2D illustrates the viscosity and interfacial tension of viscoelastic surfactants. Analogously, viscoelastic surfactants manifest a pronounced upswing in viscosity with increasing concentration. Within the concentration range of 0.01 wt% to 0.3 wt%, their interfacial tension with kerosene significantly diminishes, and in the range of 0.3 wt% to 1.0 wt%, their ability to reduce interfacial tension remains relatively stable.

Table 1 encapsulates the measured viscosity and interfacial tension of various chemical agents. Derived from these findings, we gain insights into the distribution of interfacial forces and viscosity pertaining to four distinct categories of oil displacement chemicals. In subsequent simulation endeavors, data with a chemical concentration of 0.1 wt% will be selected for in-depth exploration. This concentration was chosen because it represents a typical range in laboratory EOR studies, where different surfactant systems already show characteristic behaviors of viscosity enhancement and IFT reduction, providing a representative basis for comparison in simulations. To deepen our comprehension of the contribution mechanisms of current chemical systems to the optimal oil-water flow ratio in heavy oil, alongside the viscosity and interfacial activity of diverse chemical systems in augmenting oil recovery, it is imperative to undertake meticulous investigations of oil displacement mechanisms for varied chemical systems under diverse wetting conditions through microscopic migration processes.

**TABLE 1:** Viscosity and interfacial tension of different surfactants at varying concentrations.

Concentration (wt%)	Surfactant type	IFT (mN/m)	Viscosity (mPa·s)
0.05	Low molecule	4.57-27.14	1.05–1.52
	Thickener	29.21	27.12
	Polymer	15.04-18.74	14.32–28.54
	Viscoelastic	1.24	5.89
0.10	Low molecule	1.01–22.54	1.08–1.54
	Thickener	28.47	97.43
	Polymer	11.67–15.67	67.5–85.54
	Viscoelastic	0.75	8.47
0.30	Low molecule	0.46–14.12	1.10–1.55
	Thickener	27.54	243.23
	Polymer	8.17–12.34	141.27–148.41
	Viscoelastic	0.08	20.02

IFT, interfacial tension; viscosity refers to dynamic viscosity; kerosene was used as the oil phase. All measurements were conducted at 25 °C.

## 3 Numerical methods

### 3.1 Oil-water two-phase flow dynamics

In this work, the simulation input values of viscosity and interfacial tension were directly taken from the experimental dataset at 0.1 wt% concentration. In this paper, Navier-Stokes equations are used to describe the oil-water two-phase flow in Eulerian framework, VOF method is used to track the spatial distribution of the oil and water and the contact angle is employed for characterizing the wettability of the reservoir rock.

#### 3.1.1 Conservation equation

The differential form of the mass conservation equation for incompressible oil–water two-phase flow is given by Equation 1.

∇·u=0 (1)

where **u** is average velocity of oil phase and water phase, m·s^-1^.

#### 3.1.2 Momentum conservation equation

The conservative form of the momentum conservation equation for the oil–water two-phase flow is given by Equation 2.

$$\frac{\partial \rho u}{\partial t}+\nabla \cdot \left(\rho \text{uu}\right)-\nabla \cdot \left(\mu \tau \right)=-\nabla p+\rho g+F_{\sigma }$$ (2)

where *ρ* is average density of oil phase and water phase, kg·m^-3^; *μ* is average dynamic viscosity of the two phases, Pa·s; *p* is dynamic pressure, Pa; **g** is acceleration of gravity, m·s^-2^; **F**_*σ*_ is the interfacial tension (IFT) between oil and water, kg·m^-2^·s^-2^; **τ** is the rate of strain tensor, s^-1^, which is defined by Equation 3.

$$\tau =\left(\nabla u+{\left(\nabla u\right)}^{T}\right)$$ (3)

#### 3.1.3 Interfacial tension

The last term **F**_σ_ on the right-hand side of Equation 1 represents the interfacial tension between oil and water, which is expressed by Equation 4.

$$F_{\sigma }=\sigma \delta_{s}kn$$ (4)

where σ is the surface tension coefficient, N·m^-1^; $\delta_{s}$ is the area of oil-water interface per unit volume, m^-1^, as defined in Equation 5.

$$\delta_{s}=\left|\nabla \alpha \right|$$ (5)

where α is the volume fraction of water phase. The unit normal vector of the interface, **n**, is defined by Equation 6.

$$n=\frac{\nabla \alpha }{\left.\left|\nabla \right.\alpha \right|}$$ (6)

*k* is the curvature of the oil–water interface (*m* ^−1^), given by Equation 7.

k=∇·n (7)

#### 3.1.4 Wettability

The contact angle is commonly employed to characterize the wettability of the reservoir rock. The wettability of the homogeneous oil reservoir rock can be classified into three regimes based on different water contact angles: hydrophilicity (water-wet), intermediate-wettability and hydrophobicity (oil-wet). The wettability of the pore-scale oil reservoir rock is of great importance to accurately simulate the oil-water two-phase flow and predict the distribution of the residual oil. The contact angle of the rock is affected by the surface roughness, the composition of the rock and the thickness of the water film. It is difficult to accurately determine the contact angle of realistic reservoir rock considering the mixed wettability and the variation of the contact angle with spatial locations. And the wettability regimes of oil-wettability, intermediate-wettability and oil-wettability are considered by adjusting the value of the contact angle in this paper. In this study, wettability was incorporated into the capillary model by adjusting the contact angle, which directly controls capillary entry pressure and the configuration of fluid interfaces. Three representative contact angles were selected: 15° for strongly water-wet, 90° for neutral-wet, and 165° for strongly oil-wet conditions. These values were chosen to approximate extreme and intermediate wetting states, ensuring that the model captured the full spectrum of reservoir wettability scenarios.

In order to model the wettability of the rock, the contact angle is imposed as a boundary condition, and the unit vector normal to the interface needs to be modified according to Equation 8.

$$n=n_{w}\cos\theta +s_{w}\sin\theta$$ (8)

where **n**_*w*_ is the unit vector normal to the wall; **s**_*w*_ is the unit vector perpendicular to the contact line, tangent to and pointing into wetting-solid interface surface; *θ* is the contact angle, radian.

### 3.2 Micro characteristics of residual oil after water flooding

**FIGURE 3:**
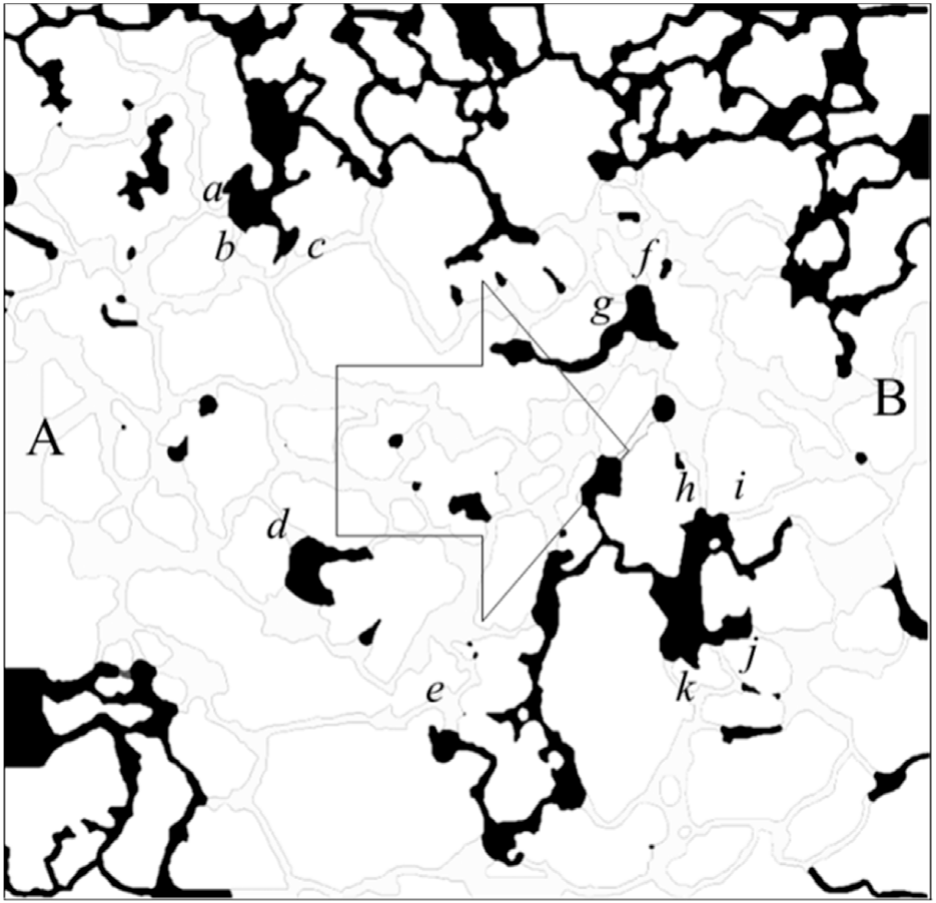
Distribution of residual oil after water flooding under the water-wet conditions.

Figure 3 illustrates the residual oil distribution following water flooding under water-wet conditions. Initially, the pores are saturated with oil, with point A serving as the injection site, point B as the effluent point, and all other boundaries functioning as impermeable walls. Water is continuously introduced at point A until no further oil production is observed at the outlet. The boundary conditions of velocity and pressure are defined in Equations 11, 12. Specifically, a fixed velocity boundary was applied at the inlet, a fixed pressure boundary at the outlet, and no-flow impermeable conditions at the lateral walls. Throughout the simulation, the oil-to-water viscosity ratio is maintained at 4:1, the oil-water interfacial tension is set at 0.05 N/m, and the equilibrium wetting angle for the water phase is 45°. The graphical representation indicates that the oil-water interface predominantly resides at the juncture between the throat and the pore (denoted by the oil-water interface labeled as a-k in the figure). The sustained position of the interface at this location is attributed to a shift in the state of capillary forces in this region.

**FIGURE 4:**
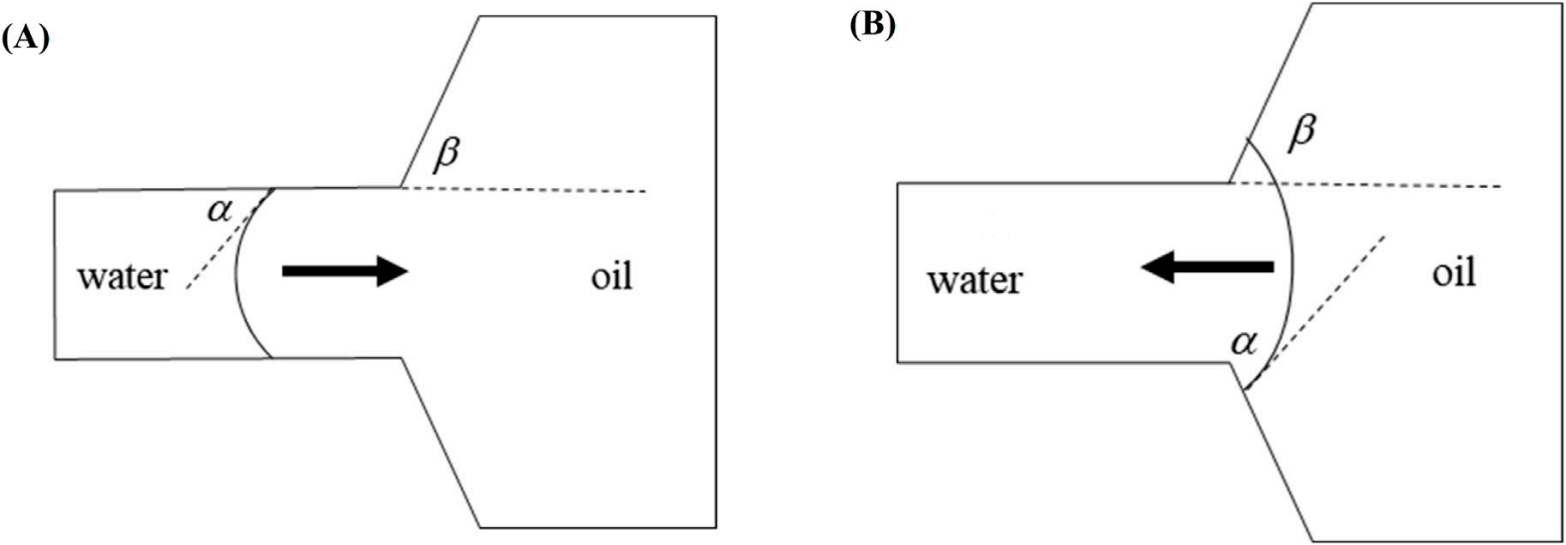
Oil-water interface: **(A)** oil-water interface in a throat, **(B)** oil-water interface in a pore.

Figure 4 illustrates the morphological configurations of the oil-water interface at distinct locations within the pore-throat conceptual model: (a) depicting the scenario where the oil-water interface resides within the throat, and (b) illustrating the scenario where the oil-water interface is positioned within the pore. In the figure, α denotes the water phase contact angle, β represents the angle formed between the throat outlet and the pore wall (referred to as the opening angle), and the black arrows symbolize the direction of capillary forces. Specifically, water is localized on the left side of the pore-throat, while oil occupies the right side.

When the oil-water interface resides within the throat, capillary forces can be delineated by the Laplace equation:

$$f=\frac{\sigma \cos\left(\alpha \right)}{R}$$ (9)

Where *f* is the horizontal capillary force, N; *σ* is the interfacial tension coefficient between oil and water, N/m; *R* is the throat radius, m. At *α* < 90^o^ (under the water-wet conditions), the value calculated according to Formula 9 is positive, and the capillary force is a dynamic force; at *α* = 90^o^ (under the neutral wet conditions), the capillary force calculated according to Formula 9 is 0; at *α* > 90^o^ (under the oil-wet conditions), the value calculated according to Formula 9 is negative, and the capillary force is a drag force.

When the oil-water interface is in the pores and its contact angle with the pore wall still is *α*, the capillary force is represented by the following equation (Hu et al., 2022; Ning et al., 2021):

$$f=\frac{\sigma \cos\left(\theta +\beta \right)}{r}$$ (10)

where *r* is the pore radius at the oil-water interface. At *θ + β* < 90^o^, the value calculated according to Formula 10 is positive, and the capillary force is a dynamic force; at *θ + β* = 90^o^, the capillary force calculated according to Formula 10 is 0; at *θ + β* > 90^o^, the value calculated according to Equation 10 is negative, and the capillary force is a drag force. Due to the influence of opening angle, the capillary force may still present a drag force even under the water-wet conditions. When the dynamic force is insufficient, the oil-water interface stays at the pore-throat junction.

**FIGURE 5:**
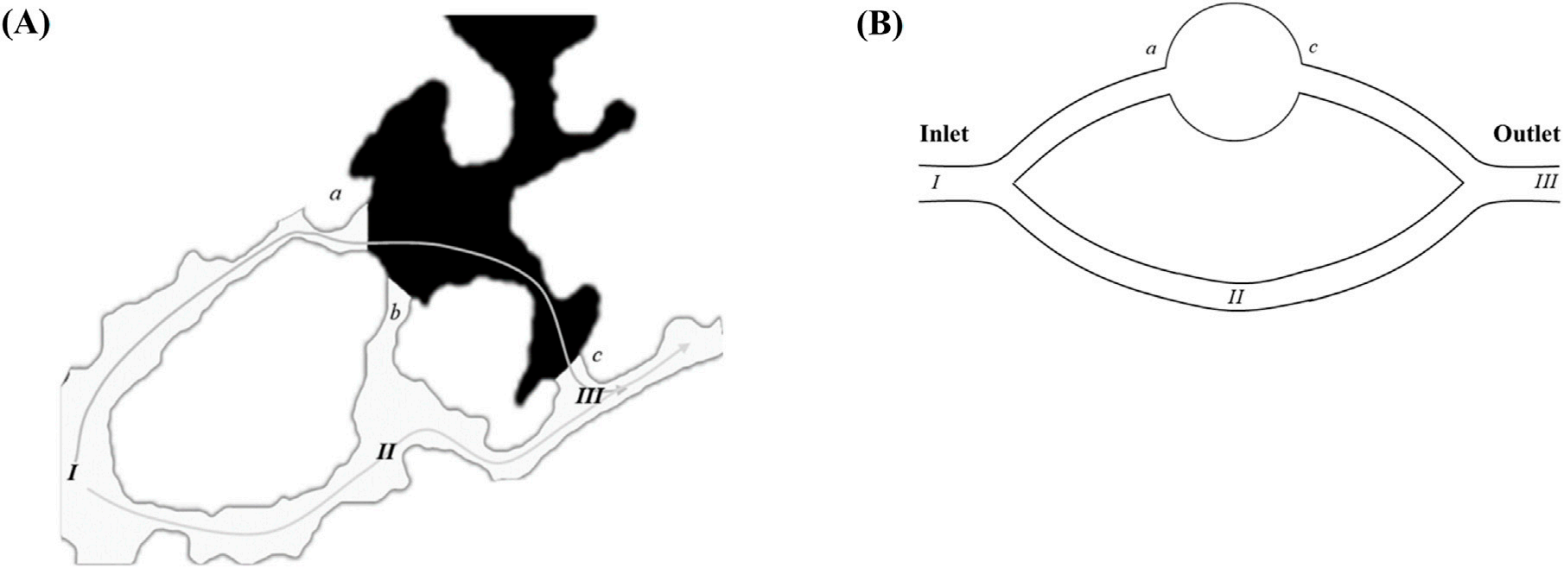
Model design: **(A)** enlarged view of residual oil distribution at the pore-throat interface (points a–c) under strongly water-wet conditions; **(B)** schematic of the parallel capillary model. The inlet and outlet positions are explicitly labeled in the schematic.

Figure 5A presents a localized magnification of the residual oil at the junction of the pore throat under water-wet conditions following water flooding. The displacement of residual oil involves two distinct paths: *I → II → III* and *I → a → b → c → III*. Leveraging insights from the paths of residual oil movement, we devised a parallel capillary model to emulate the distribution of residual oil, as depicted in Figure 5B. The parallel capillary model encompasses upper and lower channels. The upper channel incorporates a circular structure to replicate pore structures, while the lower channel lacks this circular structure, representing a pure throat structure. The channel features a depth (ℎ) of 40 μm, a width (*w*) of 20 μm, and a circular part’s radius (*r*) in the upper channel of 100 μm. The model’s initial state is entirely saturated with the oil phase, designating *I* as the injection point and *III* as the outlet. Two displacement paths are delineated: one adheres to *I → II → III*, and the other follows *I → a → b → c → III*, encompassing the pore structure (a-c). The wetting properties in the simulated channels exhibit isotropy.

## 4 Results and discussion

### 4.1 Residual oil distributions

Wettability is a critical parameter influencing the configuration of immiscible fluids within porous media (Hu et al., 2021). Therefore, in this study, three defined wettability boundary conditions are employed for simulation research: strong hydrophilic (displacing fluid with a wetting angle of 15°), neutral (displacing fluid with a wetting angle of 90°), and strong oil-wet (displacing fluid with a wetting angle of 165°).

In the process of residual oil displacement, the immiscible two-phase displacement is typically characterized by two dimensionless parameters: the capillary number (*Ca*) and the viscosity ratio (Lenormand, 1990; Wang et al., 2020). The capillary number (*Ca*) is a dimensionless quantity that indicates the ratio of viscous forces experienced by the displaced phase (oil) to capillary forces, and it is expressed by Equation 11.

$$Ca=\frac{\mu_{o}u}{\sigma }$$ (11)

where *Ca* is capillary number; $\mu_{o}$ is oil viscosity, Pa·s; *u* is the average displacement velocity, m/s.

Another crucial parameter is the viscosity ratio ( $r_{wo}$ ), defined as the ratio of the displacing fluid viscosity to the displaced fluid viscosity, and it is represented by Equation 12.

$$r_{wo}=\frac{\mu_{w}}{\mu_{o}}$$ (12)

where $r_{wo}$ is the viscosity ratio; $\mu_{w}$ is the water phase viscosity, Pa·s.

**FIGURE 6:**
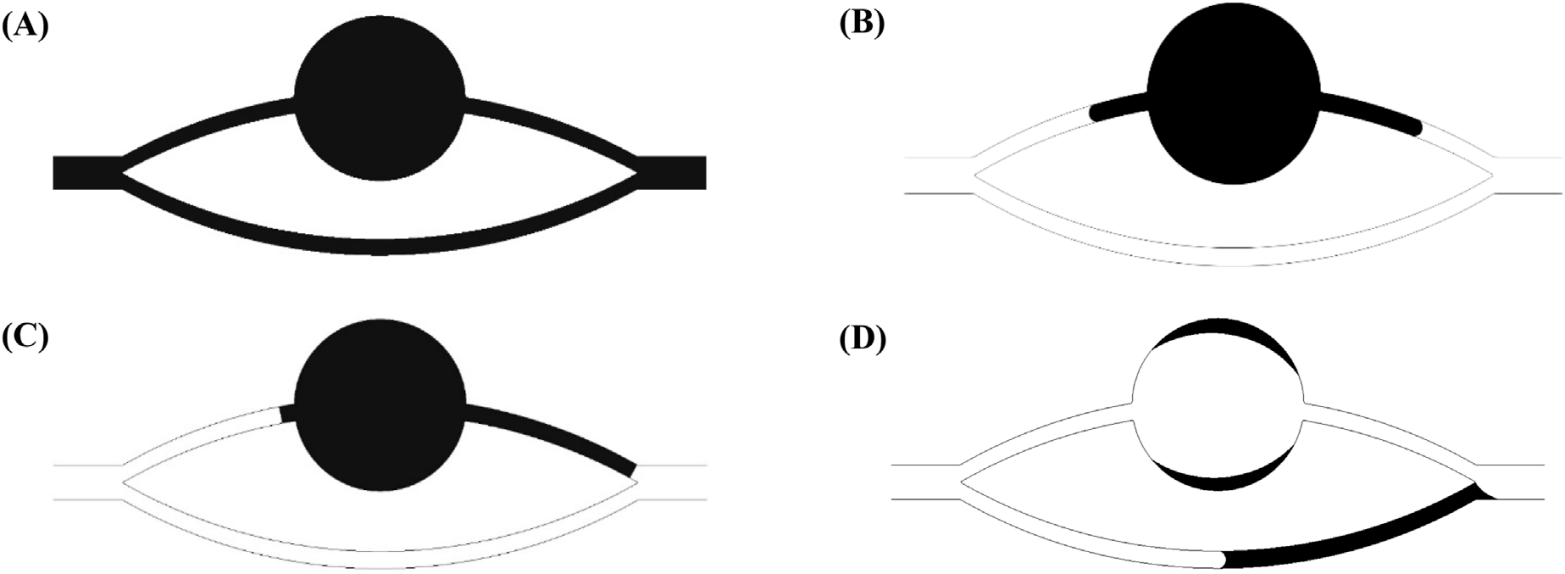
Residual oil at different stages in a parallel capillary model with different wettability (black: oil phase; white: water phase): **(A)** residual oil completely saturated; **(B)** after water flooding under the strong water-wet conditions; **(C)** after water flooding under the neutral wet conditions; **(D)** after water flooding under the strong oil-wet conditions.

Figure 6 depicts the evolution of the residual oil morphology at different temporal stages within distinct wetting conditions of parallel-tube models, with kerosene as the saturated oil phase. The graphical representation uses black for the oil phase and white for the water phase, with a water displacement velocity *u* set at 0.001 m/s.

In Figure 6A, the state is illustrated when the remaining oil saturates the channels completely. Figure 6B showcases the morphology of the residual oil following water displacement under conditions of strong water wetting. It is evident that the lower channel is fully mobilized, while the upper channel experiences comparatively lower mobilization. This discrepancy arises from the presence of a pore within the middle of the upper channel (*I → a → b → c → III*) with relatively low resistance, resulting in a higher flow rate in the upper channel compared to the lower channel (*I → II → III*). As the interface progresses to the entrance of the pore in the upper channel (i.e., point c), capillary resistance induces a stagnation of the liquid surface, enhancing the flow rate in the lower channel. Ultimately, liquid exits from the outlet. When the pressure drop across the lower channel is smaller than the capillary resistance of the upper channel, the upper channel remains blocked, resulting in residual oil entrapment. Such abrupt interface stagnation is conceptually consistent with Haines jumps observed in pore-scale experiments, where capillary and elastic effects cause sudden meniscus reconfigurations and local trapping (Sun and Santamarina, 2019). A similar channel competition behavior has been demonstrated experimentally, where the advancement of one fluid front suppresses displacement in adjacent channels due to differential capillary thresholds and flow rate sensitivity (Xu et al., 2024).

Figure 6C exhibits the morphology of the remaining oil after water displacement under conditions of neutral wetting. The remaining oil in the lower channel is fully mobilized, whereas the utilization of the upper channel remains comparatively suboptimal, akin to the scenario observed in water-wet conditions. Figure 6D portrays the morphology of the remaining oil after water displacement under conditions of oil wetting. In this case, the lower channel is only partially utilized, while the upper channel experiences nearly complete mobilization, leaving only a small film-like residual oil in the pore. The presence of a pore within the middle of the upper channel (*I → a → b → c → III*) with relatively low resistance contributes to a larger flow rate in the upper channel compared to the lower channel (*I → II → III*). As the interface advances to the entrance of the pore in the upper channel, the larger pore size reduces capillary resistance, causing the flow velocity *V*_*1*_ in the larger channel to exceed the flow velocity *V*_*2*_ in the smaller channel. Upon flooding of the larger channel with water, a small oil column remains in the smaller channel. Additionally, owing to the strongly oil-wet surface, a film-like residual oil is generated in the pore regions of the upper channel.

### 4.2 Dynamics of residual oil production process

Residual oil forms after water flooding. In the actual oil recovery process, chemical flooding begins for further production of residual oil after water flooding. The dynamics of the residual oil production process were analyzed below.

#### 4.2.1 Water-wet and neutral-wet

**FIGURE 7:**
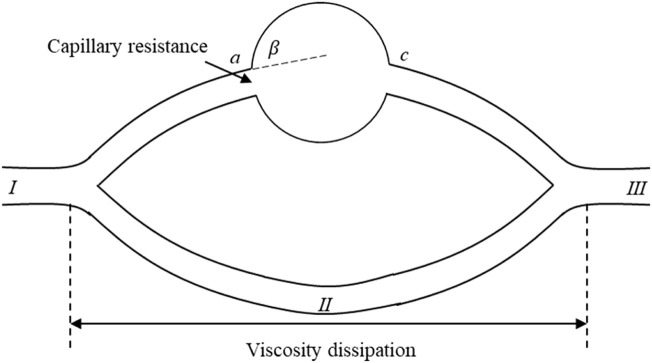
Theoretical model for residual oil production under the water-wet and neutral-wet conditions.

According to Figures 6B,C, the dynamics of further production of residual oil under the water-wet conditions and neutral wet conditions were analyzed. Figure 7 shows a theoretical model for residual oil production under the water-wet and Neutral wet Conditions. In chemical flooding and water flooding, there are two displacement channels. There is an oil-water interface in the upper channel (*I → a → b → c → III)*. According to the capillary effect mentioned earlier, the capillary force in the pores of the upper channel become a resistance at *θ* + *β* > 90^o^. The water phase in the lower channel (*I → II → III)* is a continuous phase and has no oil-water interface, so the pressure loss in the lower channel is mainly viscosity dissipation.

Based on the simulated outcomes from Figures 6B,C, we have conducted a mechanistic analysis of the enhanced mobilization of residual oil under water-wet and neutral-wet conditions. As depicted in Figure 7, a theoretical framework for the mobilization of residual oil is presented, considering both water-wet and neutral-wet scenarios. The chemical displacement process is analogous to water flooding, featuring two distinct displacement channels: upper and lower.

Within the upper channel (*I → a → b → c → III*), an oil-water interface is present. In accordance with the previously discussed capillary valve effect, when *θ* + *β* > 90^o^, the capillary forces within the pores of the upper channel transition into resistance. In the lower channel (*I → II → III*), the water phase serves as the continuous phase, eliminating the existence of an oil-water interface. Consequently, the pressure drop in the lower channel primarily arises from viscous dissipation. A detailed breakdown of this process is provided below.

When the wetting angle (*θ*) plus the geometric opening angle (*β*) surpasses 90° (*θ* + *β* > 90^o^), the capillary forces within the pores of the upper channel transition into resistance. At point a, capillary resistance is manifested, and the maximum resistance ( ${∆p}_{c,\max}$ ) is expressed as:

$${∆p}_{c,\max}=\frac{\sigma \cos\left(\theta +\beta \right)}{R}$$ (13)

where, *R* is the radius of the throat, m.

In the context of the lower channel, where it is entirely saturated with the displacing fluid, the pressure drop ( ∆p ) is defined as:

$$∆p=\frac{8\mu lu}{R^{2}}$$ (14)

When the following condition is met, there will be no residual oil in the upper channel, implying that the remaining oil in the upper part will be displaced, as expressed in Equation 15.

$$∆p>-∆p_{c,\max}$$ (15)

By substituting Equations 13, 14 into Equation 15, the resulting expression is given in Equation 16.

$$\frac{8lu\mu_{w}}{R^{2}}>-\frac{\sigma \cos\left(\theta +\beta \right)}{r}$$ (16)

#### 4.2.2 Oil-wet

**FIGURE 8:**
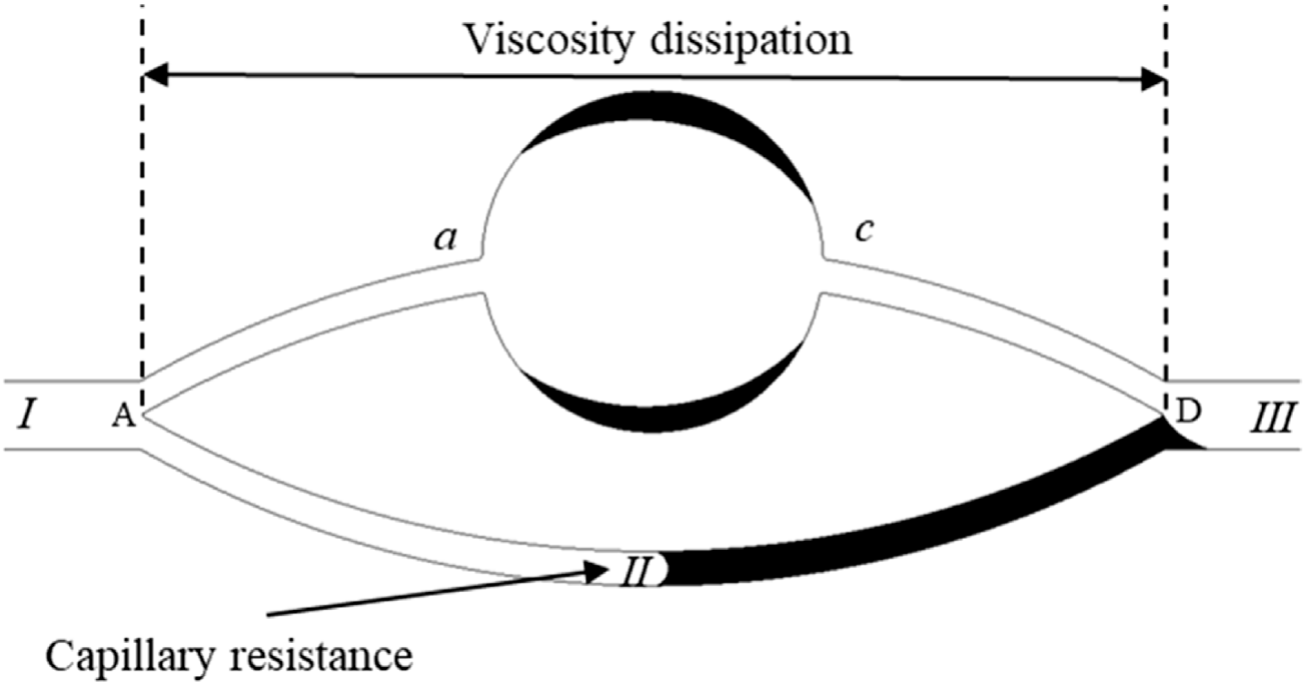
Theoretical model for residual oil production under the oil-wet conditions.

As depicted in Figure 8, the theoretical model for the mobilization of residual oil under strong oil-wet conditions is presented. Leveraging the simulation outcomes of chemical flooding, a theoretical framework is formulated to elucidate the mobilization of residual oil under the joint influence of interfacial tension and viscous forces in conditions characterized by strong oil-wettability. In the upper channel (*I → a → b → c → III*), where the water phase serves as the continuous phase, devoid of an oil-water interface, the dominant pressure loss stems from viscous dissipation. In the lower channel (*I → II → III*), featuring an oil-water interface, the hydrophobic nature of the environment induces capillary resistance at the oil-water interface, rendering the forces provided by capillarity to act as resistance. This phenomenon is illustrated in the figure below.

Based on the simulation outcomes under oil-wet conditions, it is inferred that the pressure drop in the upper channel is expressed as:

$$∆p=\frac{8\mu lu}{{\bar{r}}^{2}}$$ (17)

Where $\bar{r}$ is the equivalent pore passage radius, *l* is the length of the upper channel, *u* is the flow rate of the displacing fluid, *μ* is the displacing fluid viscosity.

Due to the oil-wet nature of the channel, the maximum resistance in the lower channel is expressed as:

$${∆p}_{c,\max}=\frac{\sigma \cos\left(\theta \right)}{R}$$ (18)

When the following equation is satisfied, the residual oil in the upper channel will be displaced, and there will be no oil remaining in the channel.

$$∆p>-∆p_{c,\max}$$ (19)

Substituting Equations 17, 18 into Equation 19 yields Equation 20.

$$\frac{\mu_{w}}{\sigma }>-\frac{{\bar{r}}^{2}\cos\left(\theta \right)}{8Rlu}$$ (20)

### 4.3 Phase diagram of residual oil production

**FIGURE 9:**
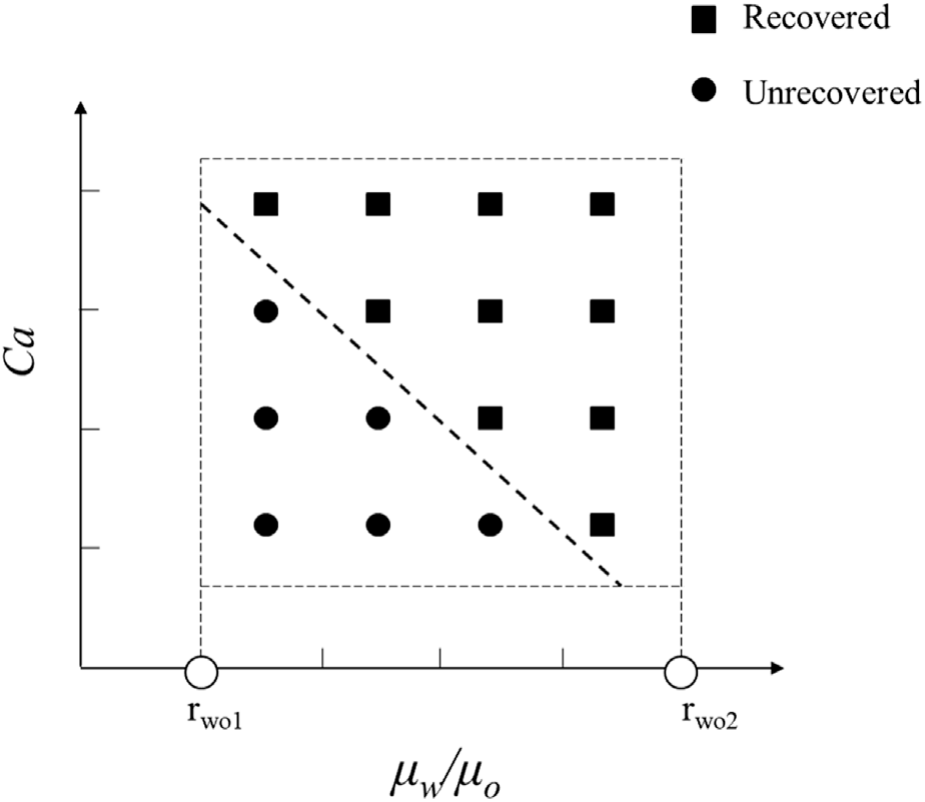
Phase diagram of capillary number and water-oil viscosity ratio in displacement, delineating boundaries between non-mobilized and mobilized residual oil zones.

In order to explore the optimal oil-water mobility ratio in the current chemical system and understand the contributions of different chemical system viscosities and activities to enhance oil recovery, this study generated a phase diagram illustrating the relationship between capillary numbers and water-oil viscosity ratios. The diagram aims to establish chemical agent property boundaries for both non-mobilized and mobilized residual oil after water flooding, as illustrated in Figure 9. Detailed analyses for different wettability conditions will be presented in the following sections.

#### 4.3.1 The thresholds for residual oil production under the strong water-wet conditions

**FIGURE 10:**
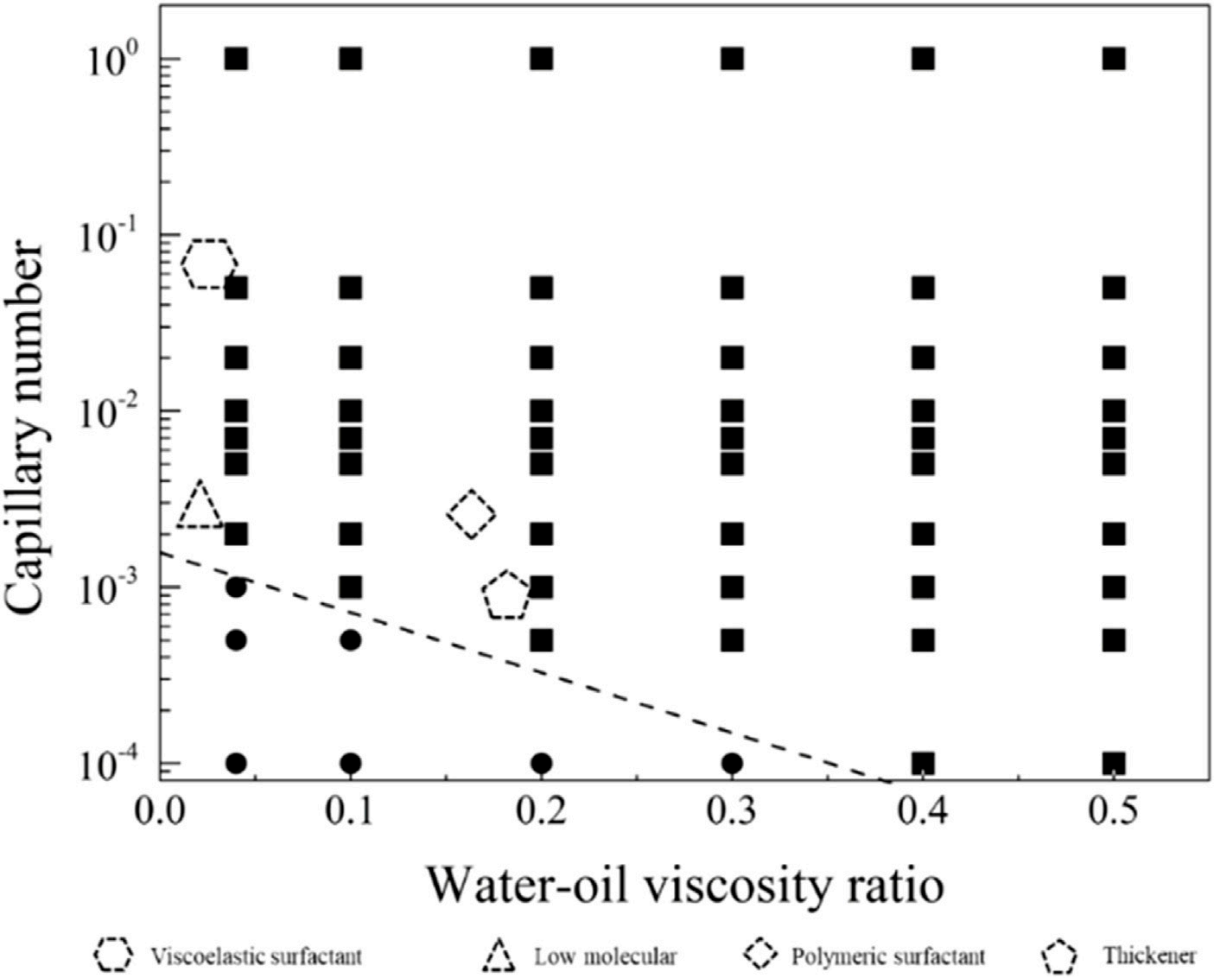
Phase diagram for displacement under the strong water-wet conditions.

**FIGURE 11:**
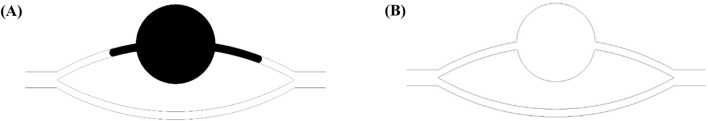
Residual oil distribution: **(A)** no production after water flooding and chemical flooding; **(B)** after chemical flood.

Figure 10 displays the phase diagram for intense water-wet displacement, encompassing 66 simulation sets under strong water-wet conditions. In Figure 11, the distribution of residual oil under these conditions is depicted. Notably, chemical agents at a concentration of 0.1 wt%, with a flow velocity of 0.001 m/s, effectively mobilized the entire residual oil. Conversely, when both the capillary number (*Ca*) and viscosity ratio ( $r_{wo}$ ) were relatively small, no mobilization of residual oil was observed. The mobilization condition boundary formula was derived through a fitting analysis of the simulation results, as given in Equation 21.

$$\mathrm{lg}\frac{\mu_{w}u}{\sigma }=-3.40324\;\frac{\mu_{w}}{\mu_{o}}-2.84885$$ (21)

where *σ* is the interfacial tension between the displaced phase and the displaced phase, u is displacement velocity, $\mu_{w}$ is displacing fluid viscosity, and $\mu_{o}$ is the displaced fluid viscosity.

By integrating this formula with the phase diagram, it becomes evident that residual oil can be effectively mobilized when the condition $\mathrm{lg}\frac{\mu_{w}u}{\sigma }>-3.40324\;\frac{\mu_{w}}{\mu_{o}}-2.84885$ is met.

**FIGURE 12:**
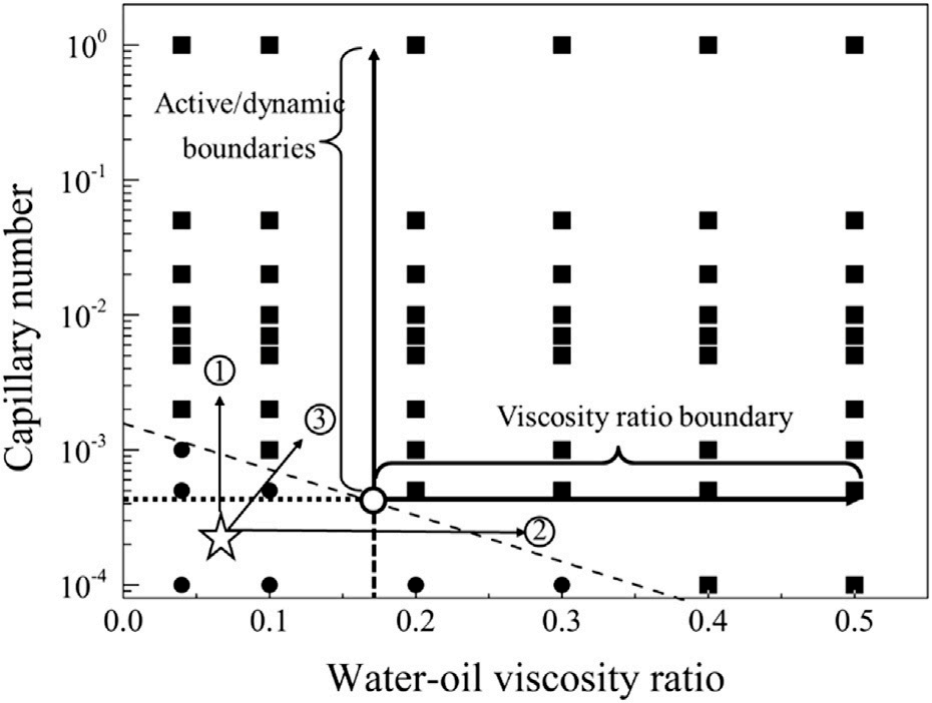
Phase diagram for displacement under the strong water-wet conditions for path selection. The star symbol represents an initial condition with residual oil in pore space, and the three arrows represent the potential routes to recovery the residual oil.

The displacement phase diagram under conditions of strong water-wettability reveals that a displacing fluid with lower viscosity necessitates heightened interfacial activity. Furthermore, based on the phase diagram, it is apparent that there are three regulatory pathways for mobilizing residual oil, as delineated in Figure 12. These pathways encompass adjustments towards diminishing interfacial tension, augmenting viscosity, and concurrently enhancing viscosity while diminishing interfacial tension.

#### 4.3.2 The thresholds for residual oil production under the neutral-wet conditions

**FIGURE 13:**
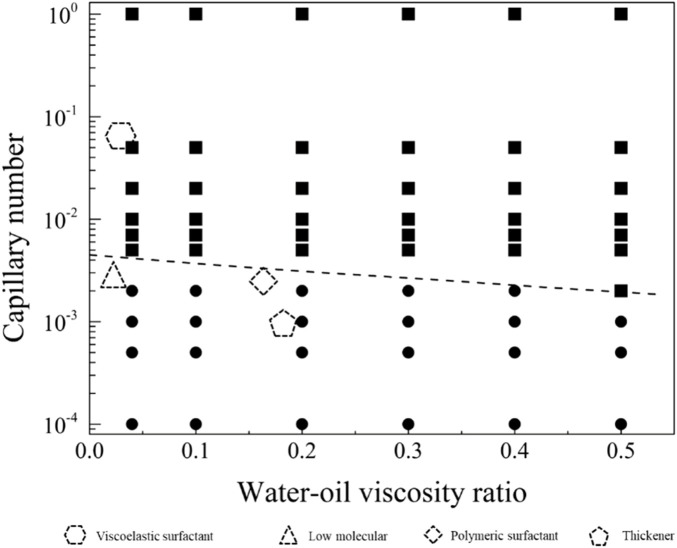
Phase diagram for displacement under the neutral wet conditions.

**FIGURE 14:**
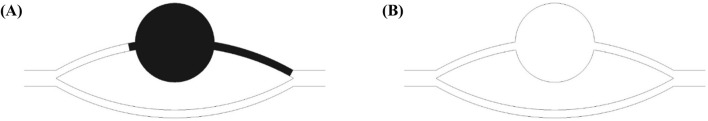
Distribution of residual oil under the neutral-wet conditions: **(A)** no production; **(B)** production.

In the neutral-wetting displacement phase diagram presented in Figure 13, and the residual oil distribution state under neutral-wetting conditions depicted in Figure 14, 66 simulations were conducted. Notably, at a flow rate of 0.001 m/s under neutral-wetting conditions, only the viscoelastic surfactant with a concentration of 0.1 wt% achieved complete displacement of the residual oil. In contrast, low-molecular-weight surfactants, thickening agents, and high-molecular-weight surfactants failed to displace the residual oil. Through a meticulous analysis of the simulation results, a phase diagram was formulated, and the boundary conditions for displacement were derived, as expressed in Equation 22.

$$\mathrm{lg}\frac{\mu_{w}u}{\sigma }=-0.75807\frac{\mu_{w}}{\mu_{o}}-2.34997$$ (22)

By integrating this formula with the phase diagram, it becomes evident that residual oil can be effectively mobilized when the condition $\mathrm{lg}\frac{\mu_{w}u}{\sigma }>-0.75807\frac{\mu_{w}}{\mu_{o}}-2.34997$ is met.

**FIGURE 15:**
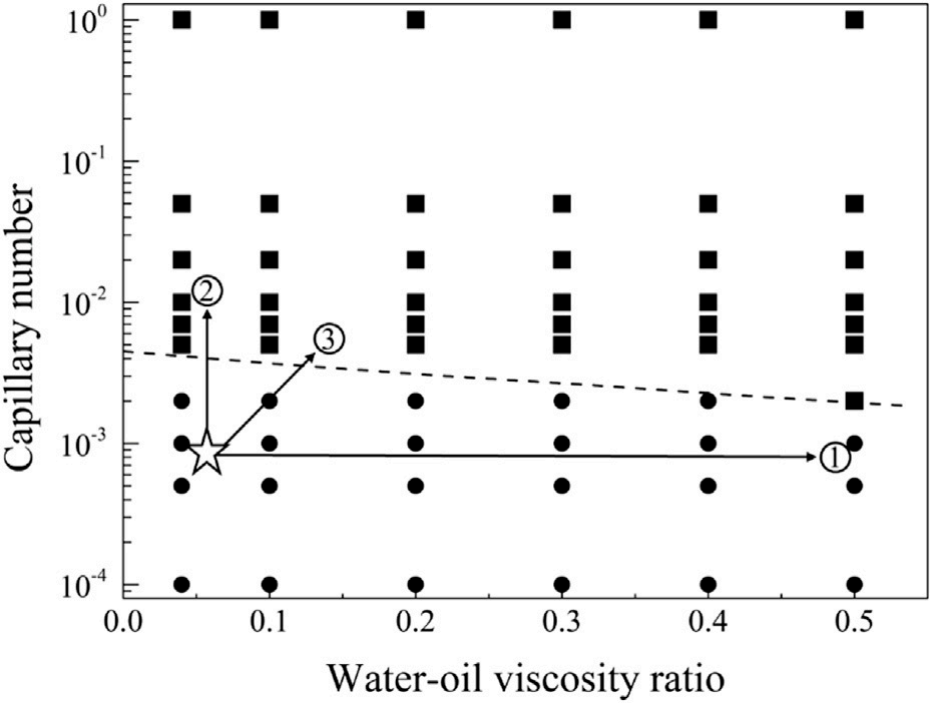
Phase diagram for displacement under the neutral wet conditions for path selection. The star symbol represents an initial condition with residual oil in pore space, and the three arrows represent the potential routes to recovery the residual oil.

The displacement phase diagram under neutral wetting conditions, as depicted in Figure 15, reveals that there is no decrease in interfacial activity requirements with the escalation of viscosity. As indicated by the phase diagram, three regulatory pathways for the mobilization of residual oil are illustrated in Figure 9. These pathways involve regulation in the directions of diminishing interfacial tension, augmenting viscosity, and concurrently increasing viscosity while diminishing interfacial tension.

#### 4.3.3 The thresholds for residual oil production under the strong oil-wet conditions

**FIGURE 16:**
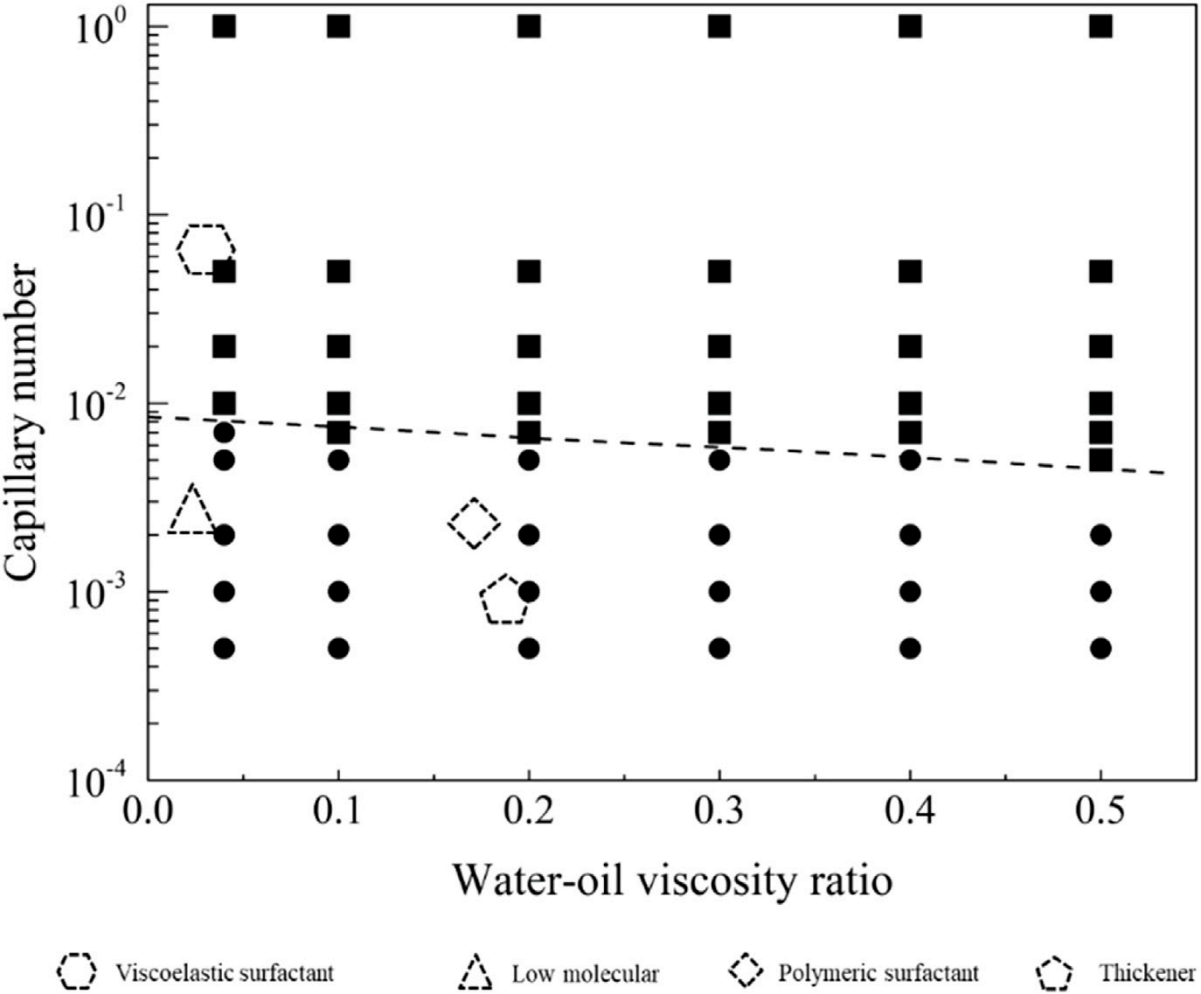
Phase diagram for displacement under the strong oil-wet conditions.

**FIGURE 17:**
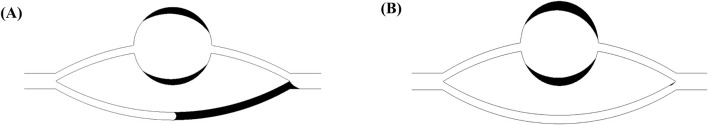
Distribution of residual oil under the strong oil-wet conditions: **(A)** no production; **(B)** production.

Figure 16 illustrates the phase diagram for strong oil-wet displacement, and Figure 17 showcases the distribution of residual oil under strong oil-wet conditions. In the case of strong oil-wet conditions, 66 simulations were conducted. Notably, at a flow rate of 0.001 m/s, only viscoelastic surfactants with a concentration of 0.1 wt% have proven effective in fully mobilizing residual oil. Conversely, low molecular weight surfactants, thickening agents, and high molecular weight surfactants did not induce mobilization of residual oil. A comprehensive analysis of the simulation results facilitated the derivation of the mobilization condition boundary, as expressed in Equation 23.

$$\mathrm{lg}\frac{\mu_{w}u}{\sigma }=-0.42578\frac{\mu_{w}}{\mu_{o}}-2.17017$$ (23)

By integrating this formula with the phase diagram, it becomes evident that residual oil can be effectively mobilized when the condition $\mathrm{lg}\frac{\mu_{w}u}{\sigma }>-0.42578\frac{\mu_{w}}{\mu_{o}}-2.17017$ is met.

**FIGURE 18:**
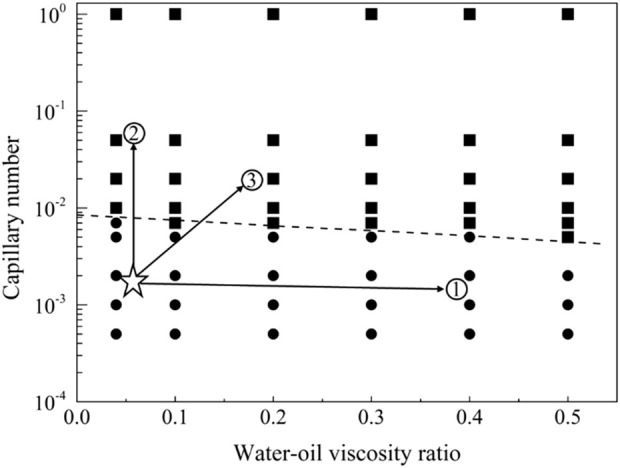
Phase diagram for displacement under the strong oil-wet conditions for path selection. The star symbol represents an initial condition with residual oil in pore space, and the three arrows represent the potential routes to recovery the residual oil.

The displacement phase diagram under strong oil-wet conditions, as depicted in Figure 18, reveals that the demand for interfacial activity does not decrease with increasing viscosity. However, an analysis of the mobilization condition boundary line in the phase diagram indicates that, for control path 1, merely altering the viscosity ratio of oil and water is insufficient to mobilize residual oil. Control path 2, involving a reduction in interfacial tension, can be effective, but the interfacial tension must decrease to 1/100 of its original value. In the case of control path 3, simultaneous increases in viscosity and interfacial activity require a fivefold increase in viscosity and a tenfold increase in interfacial activity for residual oil to be mobilized.

## 5 Conclusion

In this study, the dynamic mechanisms of water flooding and surfactant flooding were investigated using a pore–throat model and direct numerical simulation. The following main conclusions can be drawn:

1. Residual oil morphology varies with wettability. In water-wet and neutral-wet systems, the remaining oil mainly exists as connected oil ganglia within the pore body and throats, showing droplet- or cluster-like distributions without forming wall-adhered films. In contrast, under strongly oil-wet conditions, residual oil attaches to pore walls in the form of continuous films, which are more stable and considerably more difficult to mobilize.
2. Mechanistic analysis shows that the controlling factors for mobilization differ across wettability states. In water-wet and neutral-wet conditions, the displacement criterion involves both the contact angle and the pore throat opening angle, as shown in Equation 16, indicating that pore geometry strongly affects mobilization thresholds. In contrast, under strongly oil-wet conditions, the displacement expression no longer contains the pore opening angle, as shown in Equation 20, showing that mobilization is unrelated to the pore opening angle and is instead governed by interfacial properties.
3. Mobilization strategies vary according to wettability, with one optimal pathway identified for each state. In water-wet systems, reducing interfacial tension is the most effective approach. In neutral-wet systems, the best strategy is to combine viscosity enhancement with interfacial tension reduction, ensuring that viscous and capillary forces act synergistically to mobilize bridge-like oil clusters. In strongly oil-wet systems, achieving ultra-low interfacial tension is essential to detach oil films adhered to pore walls. Collectively, these insights establish viscosity and IFT thresholds as quantitative design criteria for surfactant flooding, providing a practical framework for surfactant formulation and injection strategy optimization in chemical EOR.

## Data Availability

The original contributions presented in the study are included in the article/supplementary material, further inquiries can be directed to the corresponding author.

## Nomenclature

*Ca*: Capillary number (dimensionless)
*μ*: Dynamic viscosity (Pa·s)
*σ*: Interfacial tension (N/m)
*θ*: Contact angle (°)
*β*: Opening angle of pore throat (°)
*r*: Pore radius (m)
*M*: Viscosity ratio (*μ*_w_/*μ*_o_, dimensionless)
*u*: Displacement velocity (m/s)
*p*: Pressure (Pa)
*Δp*: Pressure drop (Pa)
*l*: Channel length (m)
*R*: Throat radius (m)
***P***_***c***_: Capillary pressure (Pa)
***S***_***w***_: Water saturation (fraction)
***S***_***o***_: Oil saturation (fraction)
